# Self-perceived far vision quality and night driving difficulties are not related to night myopia or visual acuity

**DOI:** 10.1371/journal.pone.0339372

**Published:** 2026-02-05

**Authors:** Andrés Gené-Sampedro, Javier Gene-Morales, Francisco Alonso, Susana Montecelo Salvado, Sergio A. Useche

**Affiliations:** 1 INTRAS (Research Institute on Traffic and Road Safety), University of Valencia, Valencia, Spain; 2 Department of Optics, & Optometry & Vision Science, University of Valencia, Burjassot, Spain; 3 Prevention and Health in Exercise and Sport (PHES), Department of Physical Education and Sports, University of Valencia, Valencia, Spain; 4 Center for Neurosciences and Medical Research, Essilor International, affiliate of EssilorLuxottica, Paris, France; The Ohio State University, UNITED STATES OF AMERICA

## Abstract

This study aimed to psychometrically validate the Night Drive Questionnaire (NDQ), a new tool designed to assess self-reported far vision quality and difficulties related to night driving. Additionally, we evaluated the questionnaire’s potential for detecting and characterizing night myopia at an early stage. A total of 115 drivers, aged between 18 and 63 years (53% female), who regularly drove at night, completed the Night Drive Questionnaire (NDQ). Participants also had their refraction shift (night myopia) and visual acuity shift measured. Both refraction and visual acuity shift were calculated as the difference between the mesopic (low light) and photopic (normal light) measurements. We evaluated the 1) psychometric qualities through exploratory and confirmatory factor analyses, and 2) concurrent validity through correlations (Spearman) between the NDQ factors and night myopia and standard visual acuity shift and comparisons (Welch) between age groups, sexes, and night myopia levels. The NDQ displayed the best psychometric properties with a four-factor adjusted model, which includes: F1 – Quality of Far Vision, F2 – Difficulties in perceiving with central vision, F3 – Difficulties in perceiving with peripheral vision, and F4 – Difficulties in perceiving with the interaction of central and peripheral vision. This model demonstrated acceptable validity, consistency, and reliability indexes. Males compared to females reported fewer difficulties perceiving with central and peripheral vision and the combination of both (all *p* ≤ 0.011). There were no significant differences in driving difficulties based on age (all *p* ≥ 0.252) or night myopia groups (all *p* ≥ 0.242). Additionally, there were non-significant correlations between the factors and either night myopia or visual acuity shift (all *p* ≥ 0.092). In summary, the results indicate that the NDQ is a valid and reliable tool for measuring self-reported quality of far vision and the visual-related difficulties experienced during night driving, whether related to central, peripheral, or mixed vision. However, it is not effective in detecting or characterizing night myopia. Future studies should assess whether the NDQ is valid for identifying other visual function parameters that are more directly linked to night driving challenges.

## 1. Introduction

Thanks to decades of research, it is now well established that driving performance is multifactorial, with safety-related outcomes depending on the interaction between the driver’s cognitive, visual, and motor capacities, as well as the vehicle and the environment. [1,2]. Among these factors, vision is the primary source of information while driving, thus significantly contributing to whether safe or unsafe driving behaviors [3]. Additionally, in previous studies, the quality of our vision has been identified as a key determinant in self-imposed restrictions on nighttime driving [4].

In practical terms, driving at night poses significantly higher risks compared to driving during the day [5]. This increased danger is primarily due to reduced visibility and the need to quickly adjust to various lighting conditions [3,6–9]. The lighting while driving can be categorized into three types: photopic (well-lit), mesopic (low lighting, not completely dark), and scotopic (dark) [9]. Night driving primarily depends on mesopic vision rather than scotopic vision [9–10].

### 1.1. Typical night vision risk-related conditions

The existing literature highlights a wide range of visual conditions that may be associated with driving safety outcomes. Among them, there stand out visual field loss, night myopia, glare, and reduced mesopic visual acuity can significantly increase difficulties with night driving, varying across different contexts, degrees of severity, and stages of the life cycle. [6,10–12]. These visual impairments also raise the likelihood of being involved in a road crash, particularly at night [7].

Nevertheless, there is no clear consensus regarding the impact of photopic visual acuity on driving performance. While photopic visual acuity is commonly used in driving regulations, studies have shown that it is a poor predictor of actual driving ability [5,13]. For instance, mesopic visual acuity may be a better indicator of night driving performance [9]. Photopic and mesopic visual acuity refer to the optimum resolution capacity of the visual system under bright- or low-lighting conditions, respectively [14]. Night myopia describes the condition in which some individuals experience increased myopia under mesopic or low-light conditions, particularly during tasks such as nighttime driving [15]. The magnitude of this myopic shift varies among individuals, and empirical evidence highlights its potential impact; ranges shown in the literature lie between –4.0 and 0.4 D [7]. Although individual variability is common, myopic participants tend to exhibit greater night myopia than emmetropic individuals, while no significant differences were found between age groups or gender [15].

Defects in both central vision (fovea) and peripheral vision, as well as their interaction, can lead to difficulties in driving [16]. Central vision defects tend to cause more issues than peripheral defects, and they are more strongly associated with a decline in driving-related quality of life, as measured by the NEI-VFQ-51 [17]. However, most of the information we take in while driving comes from our peripheral vision, which is then directed to the fovea (central vision) through saccadic eye movements [1,18]. Therefore, effective driving has been increasingly claimed to depend on the proper coordination between central and peripheral vision [19].

### 1.2. The current study

Clinical evaluations have traditionally been the standard method for assessing visual abilities related to driving. However, subjective or indirect tools, such as questionnaires and reading tests, can provide a complementary approach to identify visual and attentional difficulties that may be overlooked in standardized assessments [1,8,18]. Night myopia and visual field are linked to night driving skills [16] and safety outcomes [7], and they can be assessed through various methods. Recent studies have introduced innovative techniques for measuring night myopia in clinical settings [15] and, potentially, in driving examination centers [12]. Specifically, García-Rojo et al. [12] evaluated differences in low-contrast visual acuity under mesopic versus photopic lighting conditions, simulating reduced illumination using a neutral density filter. The proposed method is simple and practical, making it well-suited for implementation in examination centers. Gené-Sampedro et al. [15], in contrast, quantified night myopia in a population of drivers by assessing changes in subjective refraction between photopic (standard daylight) and mesopic (typical nighttime driving) lighting conditions. They employed both ambient and chart-controlled illumination, all within time constraints suitable for routine clinical practice. Refraction was performed at a distance of 6.0 meters, using 0.01 D steps. The protocol, designed to optimize both dark adaptation and test reliability, consisted of three stages: (1) standard refraction under photopic conditions, (2) a 5-minute dark adaptation period under scotopic conditions, and (3) binocular spherical adjustment under mesopic conditions. They defined night myopia as the difference in the spherical component between photopic and mesopic measurements. However, to effectively relate night myopia to actual driving difficulties (e.g., perceiving with central vision, peripheral vision, or their interaction while driving during the night), self-perceived driving and own capabilities must be considered. These self-perceptions are associated with driving performance in both healthy individuals and those with visual or cognitive impairments [20,21]. Therefore, in addition to clinical methods, simple techniques for estimating night myopia or difficulties with visual perception may be valuable in identifying and characterizing visual impairments that could affect driving performance. This raises the question of whether night myopia can be detected and characterized early in driving recognition centers through a questionnaire, avoiding the need for specialized visual refraction analysis.

Based on the aforementioned, this study aimed to psychometrically validate a new questionnaire (the Night Drive Questionnaire, or NDQ) that assesses self-reported far vision quality and difficulties encountered during night driving. Additionally, we intended to evaluate the questionnaire’s ability to detect and characterize night myopia at an early stage.

To achieve the proposed study aims, and considering the available insights provided by previous research, we formulated two literature-based hypotheses: 1) the questionnaire will demonstrate a suitable psychometric structure and metrics, and 2) night myopia can be identified through the questionnaire without the need for advanced equipment.

## 2. Methods and materials

### 2.1. Study design and ethics

This is a cross-sectional observational study that aims to correlate the results of a self-reported questionnaire (the Night Drive Questionnaire [NDQ]) with the outcomes of sight examinations, with a focus on night myopia and visual acuity shift from photopic to mesopic conditions.

Regarding ethical considerations, all the procedures performed in this study were priorly assessed and approved by the Ethics Committee on Human Research of the University of Valencia (IRB Approval: UV-INV_ETICA-1534921) and followed Good Clinical Practice Rules and the Declaration of Helsinki. Anonymization was ensured by assigning unique identification numbers to each participant. Only the principal investigator had access to these identification numbers; all the rest of researchers involved, including data analysts, did not have access to the numbers, therefore, ensuring that reidentification was not possible.

The recruitment period for participants started on May 5, 2021, and ended on March 5, 2022. We used advertisements and email lists to recruit potential respondents. Recruitment modalities included study appeal to the university community, targeted associations of interest as the Red Cross, and the general population residing in the vicinity of the university. Selected candidates (see screening procedures below) were free to withdraw from the study at any time. In addition, all participants were adults (aged over 18) and provided written informed consent prior to their participation. The consent form was provided to participants at least 5 days before the commencement of the study, allowing adequate time for consideration before the initial visit, when they signed the consent.

### 2.2. Sample selection and features

Considering the population profiling required for this study, the inclusion criteria were defined as follows: participants aged 18–65 years, with best-corrected distance visual acuity of +0.10 logMAR or better, a distance spherical equivalent between −8.00 and +6.00 diopters (D), cylindrical error up to −3.00 D, anisometropia up to 1.50 D, and regular night driving (at least four days per week) were deemed eligible. These criteria reflect the study’s focus on the general driving population. In Spain, the legal minimum age for obtaining a driver’s license is 18 years. Participants older than 65 years were excluded to avoid potential confounding effects of age-related ocular conditions, such as early lens opacities, cataracts, or age-related macular degeneration. Refractive and binocular vision thresholds were set to ensure sufficient visual function and to avoid potential issues related to binocular disparity. Regular nighttime driving was required to ensure comparable exposure to low-light conditions among participants, thus supporting the reliability of between-subject comparisons.

As standardized exclusion criteria, individuals with a history of ocular surgery, ocular pathology, untreated and/or uncontrolled systemic conditions, or medical treatments that could affect vision or interfere with study variables were excluded. Additionally, participants with migraine, epilepsy, binocular vision problems, permanent use of contact lenses or tinted lenses, or specialized knowledge in optometry, ophthalmic lenses, or optics were not included in the study.

After carefully conducting such screening procedures, a total of 115 drivers (age: 34.51 ± 13.09 years, range 18–63) from both sexes (53% female; 47% male) were selected and participated in the empirical study. Regarding their driving profile, all participants held valid driving licenses and regularly drove at night, specifically on at least four days a week for a minimum of 20 minutes each day.

Most subjects (82.6%) reported driving a car, 14.8% driving a car and riding a motorbike, and 2.7% other vehicle combinations such as driving a car and a van, a motorbike and a van (0.9%), and driving a bus (0.9%). Table 1 provides a detailed overview of the participants’ basic demographic characteristics and visual self-assessments.

**Table 1:** Participants’ basic demographic and self-assessed visual characteristics.

Variable	Response	Frequency	Percentage
Driving experience	< 1 year	11	9.6
	1 to 5 years	19	16.5
	5 to 10 years	20	17.4
	10 to 15 years	21	18.3
	> 15 years	44	38.3
Far vision quality in general	Very poor	0	0
	Poor	4	3.5
	Acceptable	35	30.4
	Good	64	55.7
	Excellent	12	10.4
Far vision quality during day driving	Very poor	0	0
	Poor	2	1.7
	Acceptable	22	19.1
	Good	74	64.3
	Excellent	17	14.8
Far vision quality during night driving^a^	Very poor	1	0.9
	Poor	26	22.6
	Acceptable	57	49.6
	Good	30	6.1
	Excellent	1	0.9

^a^Night refers to the time lapse between 7:00 pm and 7:00 am.

### 2.3. Procedure

**Fig 1:**
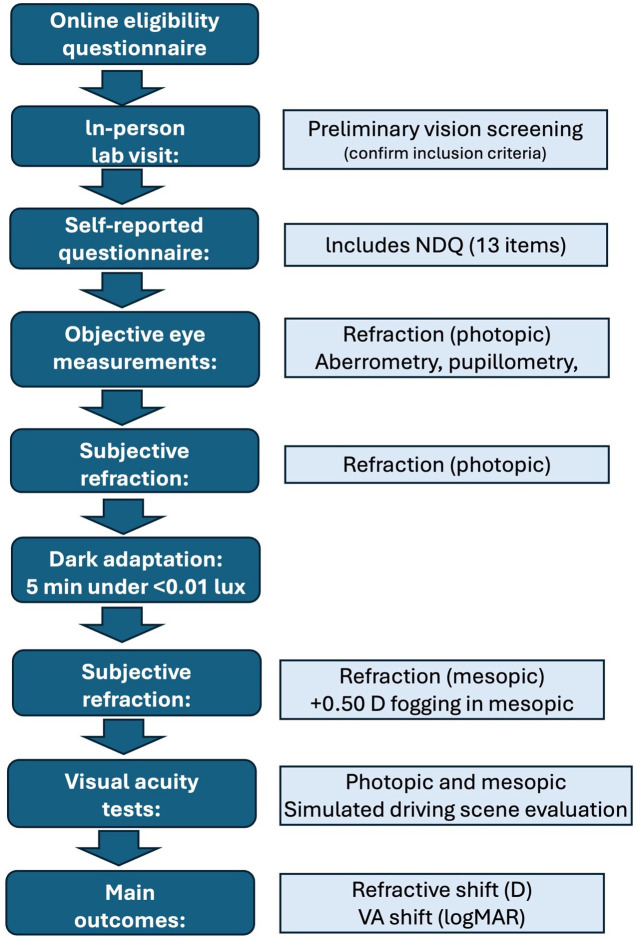
Flow chart of study procedures. D: diopters; logMAR: logarithm of the minimum angle of resolution; NDQ: night drive questionnaire; VA: visual acuity.

Participants first completed an online questionnaire to determine their eligibility. After this, they attended a single session in the laboratory where they underwent a preliminary vision examination to assess inclusion criteria. Following the examination, they answered an electronic questionnaire that included the NDQ. Then, they underwent sight examination under photopic and mesopic conditions. Each procedure can be found in Fig 1 and is detailed in the subsections that follow. The total duration of the session was approximately 45 minutes.

#### 2.3.1. Sight examination.

All measurements were conducted by the same specialists following the protocol established in a previous validation study by our research group [15]. The initial vision examination included evaluating best-corrected distance visual acuity, binocular vision screening (cover/uncover test and vergence ability), stereo acuity, and ocular motility. Then, we measured objective refraction, ocular aberrometry, and pupillometry using a multifunctional eye diagnostic device, specifically the Wave Analyzer Medica 700 (Essilor, France).

For the subjective refraction, we conducted two measurements (photopic and mesopic) at 6.0 meters using an automated phoropter (VisionR-^TM^800, Essilor, France), which allows resolution of 0.01D steps by continuous power lens variation [22]. The procedure included the following steps: 1. Photopic conditions (lightroom: 45 lux) 2. Dark adaptation 5 minutes (dark room < 0.01 lux) 3. Mesopic conditions (lightroom: 0.01 lux). Spectral illuminance (lux) measurements were taken at eye level with an illuminance spectrophotometer (Konica Minolta CL-200, Prolite-Ltd, UK).

Randomized ETDRS charts were used to prevent learning effects during visual acuity assessments. The Vision-C 600 (Essilor, France) screen displayed the charts at a photopic level of 181 cd/m² and a mesopic level of 1.1 cd/m², with letter contrast above 90%. Luminance was evaluated with a ColorCAL MLII Colorimeter (Cambridge Research Systems, UK). For detailed steps of the refraction protocol, refer to Gené-Sampedro et al. [15].

We started with the standard subjective refraction in photopic conditions, utilizing objective refraction as our starting point. Then applied monocular fogging/defogging, cross-cylinder techniques, and performed biocular and binocular balance assessments. The most positive refraction that provided the best visual acuity (MPMVA) was determined for both monocular and binocular conditions. After a 5-minute dark adaptation (using occluders and no room light), we applied +0.5 D fogging for the mesopic condition. Mesopic refraction was performed directly under binocular conditions, adjusting only the spherical component (without modifying cylinder), to better simulate real-world low-light environments. This methodology is consistent with previous research [15,23] that demonstrated that night myopia tends to be reduced under binocular conditions compared to monocular ones. The refractive shift was calculated as the difference between these two binocular measurements. The MPMVA was adjusted based on photopic values without changing the astigmatic component. Perceptual appreciation (PA) determined the endpoint of mesopic refraction, with adjustments of 0.12 D made for visual comfort as outlined in Gené-Sampedro et al. [15]. Participants compared the MPMVA with –0.25 D and rated the difference as slight, moderate, or significant. Finally, they evaluated photopic and mesopic refractions in a simulated night-driving scene and graded their quality of vision preference.

The outcomes of the vision examination used for this study were the night myopia (i.e., refractive shift) and visual acuity shift:

1) *Refractive shift:* This refers to the difference in binocular sphere between night (mesopic) refraction and standard (photopic) refraction, which typically results in a myopic shift.
2) *Standard visual acuity shift (in logMAR units):* This is the change in binocular visual acuity between mesopic and photopic lighting conditions while wearing the standard refraction.

#### 2.3.2. The Night Drive Questionnaire.

The questionnaire is part of a study examining age-related differences in night driving performance under night myopia and was developed following previous research on how to develop and validate scales for health, social, and behavioral research [24]. After articulating the domain to be measured, i.e., nighttime driving difficulties related to vision, and carefully reviewing validated tools such as the Vision and Night Driving Questionnaire (VND-Q) [8] and the Low Luminance Questionnaire (LLQ) [25], as well as observational data from pilot simulator sessions and literature on the visual–cognitive demands of night driving, we designed each NDQ item using both deductive and inductive methods [24]. Previous questionnaires have proven effective in targeted populations, including individuals with age-related macular degeneration (AMD) [25,26] and older drivers in closed-road assessments [27]. Additionally, preliminary content validity was confirmed through evaluation by experts and target population judges. Next steps to develop and validate the NDQ, including extraction of factors, item reduction analysis, scale evaluation, reliability, and validity, can be found in “Statistical Analysis.”

Finally, the Night Drive Questionnaire (NDQ) contained 13 multiple-choice questions (see Supplementary Material 1). The first three questions (Items 1–3) assess far vision quality, while the remaining ten questions (Items 4–13) focus on visual or vision-related difficulties encountered while driving at night. Verbal explanations were provided beforehand to ensure consistent interpretation. Participants were guided on the meaning of each term and instructed to rate based on their typical real-world driving experience.

For items 1–3, a 5-point Likert scale is used, with response options ranging from 1 (very poor) to 5 (excellent); therefore, higher scores closer to 5 indicate better vision quality (positive). For Items 4–13, another 5-point Likert scale is employed, with answers ranging from 1 (no difficulty) to 5 (extreme difficulty), meaning that scores closer to 5 suggest greater driving difficulties at night (negative).

Four factors were theoretically plausible (see Table 2): 1) quality of distance vision, 2) difficulties with central vision perception, 3) difficulties with peripheral vision perception, and 4) difficulties perceiving the interaction between central and peripheral vision.

**Table 2:** Dimensional composition of the Night Drive Questionnaire (NDQ).

Factor		Items
1	Quality of far vision	1, 2, 3
2	Difficulties in perceiving with central vision	4, 5, 6, 7
3	Difficulties in perceiving with peripheral vision	8, 9, 10
4	Difficulties in perceiving with the interaction of central and peripheral vision	11, 12, 13

### 2.4. Statistical analyses

The statistical analyses conducted in this study were performed using the latest version of the Statistical Package for Social Sciences (SPSS) software for macOS (update 29.0, IBM Corp., Armonk, NY, United States) and IBM SPSS AMOS, update 29.0. According to the study aims, the statistical analyses were divided into two main parts: first, the construct validation of the psychometric properties of the NDQ, and second, the concurrent validation of the NDQ to detect and characterize night myopia in drivers.

Before starting the validation, we performed a basic data curation, including evaluation of outliers and grouping according to sex (male and female), age (young adults [18–25 years], adults [26–50 years], and older adults [50–65 years]), and levels of night myopia (lower night myopia magnitude [≥–0.38 D], indicating less impact or milder night myopia; and higher night myopia magnitude [<–0.38 D], indicating greater impact or more pronounced myopia). The cutoffs for the age groups were based on Medical Subject Headings (MeSH) and adapted to our sample distribution, and the night myopia groups were formed according to the sample median.

At this point, we started the validation procedures following standard psychometric validation procedures [24]. First, we verified the adequacy of the dataset for factor analysis using the Kaiser–Meyer–Olkin (KMO) measure of sampling adequacy and Bartlett’s test of sphericity. Sampling adequacy was considered acceptable for KMO values > 0.70, very good for KMO > 0.080, and excellent for KMO > 0.90; Bartlett’s test was expected to reach statistical significance (*p* < 0.05). Once adequacy was confirmed, we evaluated factorial distribution of the NDQ using exploratory factor analysis (EFA) and confirmatory factor analysis (CFA) using consecutive fit steps (forward). Data was standardized to set factor loadings to the same scale and, therefore, be able to compare the size of each factor loading [28,29]. We chose CFA considering its benefits in terms of management of ordinal, non-normally distributed data [30,31] and considering that it allows the evaluation of diverse models under diverse theoretical suppositions. We used as estimators to weigh the model fit the Chi-square (*χ*^*2*^), discrepancy ratio (CMIN/df or χ^2^/df), Root Mean Square Error of Approximation (RMSEA), and the following ordinal indexes: Tucket-Lewis Index (TLI), Normed Fit Index (NFI), Incremental Fit Index (IFI), and Confirmatory Fit Index (CFI) [32]. Goodness-of-fit cutoff points were established following previous expert literature [32]: CFI/NFI indexes > 0.90, an RMSEA < 0.080, and a χ^2^/df ratio < 5.0.

Second, we evaluated the suitability of the model through the strength and coherence of the estimates and the absence of high levels of redundant modification indexes [33]. The internal consistency and reliability of the NDQ items were calculated by means of Cronbach’s α coefficients and Composite Reliability Indexes (CRIs), which are calculated from the factor loadings and residuals obtained in the CFA. This indicator helps to overcome some of the traditional limitations of Cronbach’s Alpha coefficients as a single way of determining the reliability (or not) of a questionnaire scale [34,35]. The factor loadings were graphically represented, including the correlation between each of the factors. To assess convergent and discriminant validity, we calculated the Average Variance Extracted (AVE) and examined the Fornell–Larcker criterion for each factor of the NDQ. Convergent validity was considered adequate when AVE ≥ 0.50 and discriminant validity when the square root of the AVE for each factor exceeded its correlations with the other factors. These analyses were conducted based on the standardized loadings obtained in the confirmatory factor analysis (CFA). Finally, inter-item correlations and corrected item–total correlations (CITC) were calculated to assess the internal homogeneity of each factor. We reported the range of inter-item correlations, CITC values, and Cronbach’s α if the item was deleted.

As for the second part, we evaluated the concurrent known-groups validity of the NDQ to provide different outcomes between sexes and age groups and detect and characterize night myopia. For such purpose, we assessed Spearman’s *rho* 2-tailed correlations between the factors of the NDQ and both the night myopia and standard visual acuity shift. Additionally, we conducted between-group comparisons for sex, age, and night myopia using the Welch test, which is a Student’s t-based nonparametric statistical technique more appropriate than a parametric analysis of variance (ANOVA) when variances are unequal and sample sizes are not proportional. The effect sizes were calculated with eta squared (η^2^), with η^2^ ≤ 0.06 being a small effect, 0.06 < η^2^ ≤ 0.14 a medium effect, and η^2^ > 0.14 a large effect. Post-hoc tests for age group comparisons were conducted with the Games-Howell adjustment. A cutoff criterion of *p* < 0.05 was uniformly established for correlational and comparative tests.

## 3. Results

### 3.1. Measurement model and reliability measures

Sampling adequacy for factor analysis was confirmed by a Kaiser–Meyer–Olkin (KMO) value of 0.877, which indicates a very good degree of common variance among the items. Bartlett’s test of sphericity was significant (χ²(78) = 737.55, p < 0.001), supporting the suitability of the correlation matrix for factor analysis. These results justified proceeding with the exploratory and confirmatory factor analyses.

First, we evaluated the validity of the model measured by the Night Drive Questionnaire, to understand its factorial structure. The CFA outcomes suggested a four-factor adjusted model as a solution, with 56.1% (Factor 1), 39.3% (Factor 2), 60.9% (Factor 3), and 61.1% of the variance explained, and all item factor loadings (λ coefficients) above 0.48. This can be considered an overall adequate set of goodness-of-fit indexes.

Three possible models were compared: (*i*) a bifactorial baseline model, assuming two dimensions of answers, i.e., quality of far vision and night driving difficulties, what a priori makes sense but could impair the discriminative value of a more specific multi-factorial structure; (*ii*) an unadjusted (baseline) four-factor model composed of the theoretically hypothesized dimensions appended in the Methods Section; and (*iii*) an adjusted (improved) four-factor structure considering variable and error covariances and large (and always theoretically parsimonious) modification indexes [36].

The bifactorial and four-factor unadjusted models were quite similar. However, the four-factor model’s ordinal metrics outweighed the bifactorial model and showed greater theoretical consistency. Additionally, this four-factor model was adjustable through close evaluation of psychometric improvements. In this regard, we identified large modification indexes that revealed relevant relationships between some items. Finally, the adjusted model (four factors) appropriately fitted the data, keeping qualitative sense and adequate psychometric values, as shown in Table 3.

**Table 3:** Competitive factor analysis: Goodness-of-fit indices and model adjustment.

Model	*X* ^2^	df^a^	CMIN/df^b^	*p*	RMSEA^c^	90% CI^d^		Ordinal Indexes			
						Lower	Upper	TLI^e^	NFI^f^	IFI^g^	CFI^h^
*Baseline models*											
Bifactorial	148.788	64	2.325	<0.001	0.108	0.085	0.131	0.851	0.807	0.880	0.878
Four-factor	141.026	59	2.390	<0.001	0.110	0.087	0.134	0.844	0.817	0.885	0.882
*Adjusted model*											
Four-factor (retained)	93.225	52	1.793	<0.001	0.078	0.050	0.106	0.911	0.879	0.943	0.941

*Notes:* a: df = Degrees of freedom; b: CMIN/df = Disparity ratio (X2/df); c: RMSEA = Root Mean Square Error of Approximation; d: Confidence Interval for RMSEA (α = .010); e: TLI = Tucker-Lewis Fit Index (rho2); f: NFI = Normed Fit Index (Delta1); g: IFI = Incremental Fit Index (Delta2); h: CFI = Confirmatory Fit Index.

**Fig 2:**
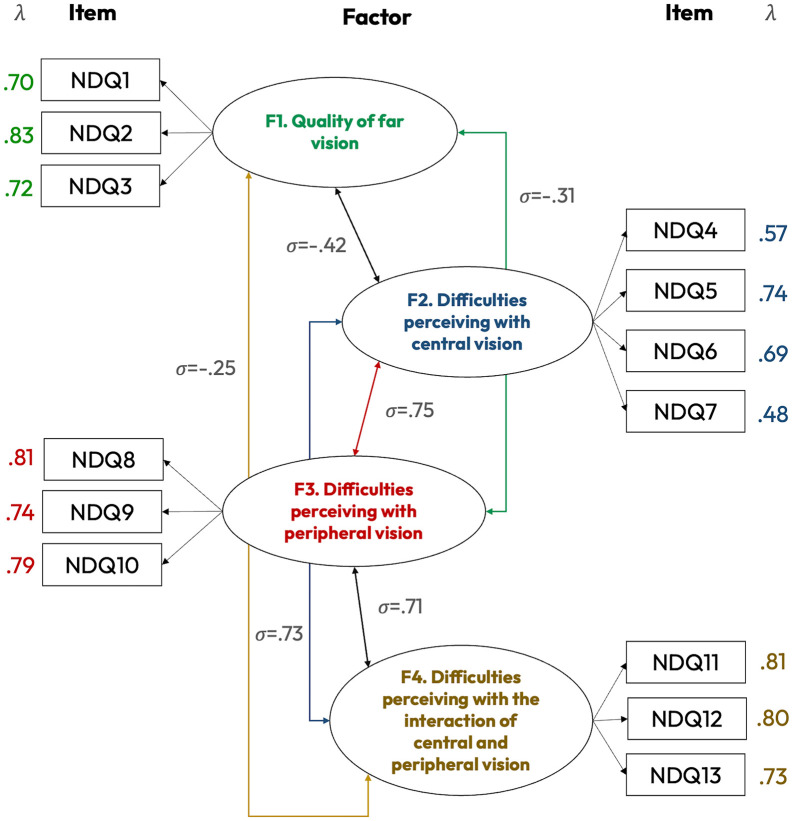
Standardized parameter estimates and factor correlations. *Note:* All standardized estimates were *p* < 0.05; questionnaire items are presented inside squares and item factors inside ellipses.

After the CFA, the validity, reliability, and consistency of each item were evaluated by means of standardized factor loadings (λ coefficients) with standard errors (SE) and critical ratios (CR). Additionally, the Composite Reliability Indexes (CRIs), Cronbach’s Alpha coefficients (α), Omegas (ω), Gutman’s Lambda-5 (λ_5_), Average Variance Extracted (AVE), Fornell–Larcker criterion evaluation, inter- item correlations, and corrected item–total correlations (CITC) were reported for each of the four factors. Tables 4 and 5 present the outcomes of these validation analyses for each item and factor composing the Night Drive Questionnaire. Additionally, Fig 2 shows the structure of the questionnaire items and factors, including correlation among factors and λ coefficients.

**Table 4:** Descriptive outcomes of the Night Drive Questionnaire (NDQ).

Item			M^b^	SD^c^	Lower Bound	Upper Bound	λ^d^	SE^e^	CR ^f^
NDQ1	Self-rated distance vision performance		3.73	0.69	3.60	3.86	0.699	0.135	6.670
NDQ2	Self-rated distance vision performance during day driving		3.92	0.64	3.80	4.04	0.825	0.163	6.689
NDQ3	Self-rated distance vision performance during night driving ^a^		3.03	0.75	2.90	3.17	0.717	0.166	6.67
NDQ4	Difficulty reading traffic signs during night driving		2.33	0.89	2.17	2.50	0.571	0.221	4.597
NDQ5	Difficulty seeing the road when it rains during night driving		2.86	0.88	2.69	3.02	0.737	0.214	6.276
NDQ6	Difficulty seeing the road due to the lights of cars that come from the front during night driving		2.80	0.99	2.62	2.99	0.685	0.237	6.106
NDQ7	Difficulty seeing the road due to the street/road lighting during night driving		1.80	0.97	1.62	1.97	0.483	0.214	4.597
NDQ8	Difficulty detecting moving objects during night driving		1.88	0.78	1.73	2.02	0.812	0.095	9.286
NDQ9	Difficulty seeing obstacles during night driving		2.18	0.93	2.01	2.36	0.738	0.128	8.448
NDQ10	Difficulty seeing obstacles that appear suddenly during night driving		1.97	0.92	1.80	2.14	0.789	0.122	9.286
NDQ11	Difficulty seeing traffic lights during night driving		1.91	0.91	1.74	2.08	0.809	0.146	7.405
NDQ12	Difficulty estimating the distance to a detour during night driving		1.88	1.01	1.70	2.07	0.800	0.104	7.687
NDQ13	Difficulty estimating the distance to a nearby vehicle during night driving		1.71	0.88	1.55	1.87	0.733	0.124	7.405
**Factors**		**M**	**SD**	**Lower Bound**	**Upper Bound**	**CRI** ^**g**^	**α** ^**h**^	**Ω** ^**i**^	**λ** ^**dj**^
F1	Quality of far vision	3.56	0.58	3.46	3.67	0.915	0.783	0.784	0.776
F2	Difficulties perceiving with central vision	2.44	0.69	2.32	2.57	0.917	0.722	0.724	0.734
F3	Difficulties perceiving with peripheral vision	2.01	0.76	1.87	2.15	0.932	0.815	0.816	0.808
F4	Difficulties perceiving with the interaction of central and peripheral vision	1.83	0.78	1.69	1.98	0.912	0.777	0.782	0.778

*Notes for the table*: Values are presented in a scale from 1 (less performance/difficulty) to 5 (more performance/difficulty).

^a^Night driving is considered between 19:00 and 7:00 hours; ^b^ mean; ^c^ standard deviation; ^d^ factor loadings; ^e^ standard error; ^f^ critical ratio; ^g^ Composite Reliability Index; ^h^ Cronbach’s Alpha; ^i^ omega; ^j^ Gutman’s Lambda-5.

**Table 5:** Internal homogeneity analysis: Inter-item correlations and corrected item–total correlations.

Factor	Cronbach’s α	Inter-item correlation range	CITC range	α if item deleted
F1. Quality of far vision	0.783	0.475–0.608	0.584–0.688	0.643–0.755
F2. Difficulties with central vision	0.722	0.216–0.524	0.382–0.631	0.582–0.737
F3. Difficulties with peripheral vision	0.815	0.544–0.681	0.614–0.720	0.705–0.805
F4. Interaction central–peripheral vision	0.777	0.450–0.622	0.553–0.692	0.619–0.763

All the factor loads were adequate (λ coefficients > 0.48). This confirms the good fit of the model and contributes to discarding the need to delete items that present potential psychometric shortcomings.

The Composite Reliability Indexes (CRIs) ranged between 0.912 and 0.932, which are considered good reliabilities for all four latent constructs. CRI for F1 (Quality of far vision) was 0.915; 0.917 for F2 (Difficulties perceiving with central vision); 0.932 for F3 (Difficulties perceiving with peripheral vision); and 0.912 for F4 (Difficulties perceiving with the interaction of central and peripheral vision).

Regarding internal consistency indexes, both Cronbach’s Alpha (α) and McDonald’s Omega (ω) coefficients were all above the minimum literature-based cut-off thresholds, which is over the usual 0.700 criteria. These coefficients ranged [0.722–0.816], indicating a suitable psychometric internal consistency, as noted in methodological sources that also suggest using Alpha coefficients as a complementary but not as a single reliability index [37,38]. The results suggest adequate internal reliability, with α = 0.783, ω = 0.784, λ_5_ = 0.776 for F1 – Quality of far vision; α = 0.722, ω = 0.724, λ_5_ = 0.734 for F2 – Difficulties perceiving with central vision; α = 0.815, ω = 0.816, λ_5_ = 0.808 for F3 – Difficulties perceiving with peripheral vision; and α = 0.777, ω = 0.782, λ_5_ = 0.778 for F4 – Difficulties perceiving with the interaction of central and peripheral vision.

Convergent validity and discriminant validity were supported in Factors 1, 3, and 4 (AVE = 0.56, 0.61, and 0.61, respectively; √AVE > inter-factor correlation [Fornell–Larcker criterion]). Although Factor 2 exhibited a lower AVE (0.39) and did not fully satisfy the Fornell–Larcker criterion with Factors 3 and 4, its high CRI (0.912), together with acceptable internal consistency indices (α = 0.722; Ω = 0.724; λ_5_ = 0.734) and theoretically grounded content, support its reliability.

Finally, inter-item correlations and corrected item–total correlations supported the internal homogeneity of the four factors. For Factor 1 (quality of far vision), α = 0.783, with inter-item correlations between 0.475 and 0.608, and CITC ranging from 0.584 to 0.688. For Factor 2 (difficulties perceiving with central vision), α = 0.722, inter-item correlations ranged from 0.216 to 0.524, and CITC from 0.382 to 0.631; deletion of item NDQ7 would have raised α to 0.737, but it was retained due to its conceptual relevance. Factor 3 (difficulties perceiving with peripheral vision) showed α = 0.815, with inter-item correlations between 0.544 and 0.681 and CITC > 0.61. Finally, Factor 4 (interaction of central and peripheral vision) yielded α = 0.777, with inter-item correlations between 0.450 and 0.622 and CITC between 0.553 and 0.692. In no other case, apart from Item 7, did the removal of items substantially improve internal consistency, therefore, indicating that all items contributed adequately to each factor. Inter-factor correlations were all < 0.85, indicating non-excessive multicollinearity.

### 3.2. Factor correlations and association with night myopia

The average night myopia (refraction shift) and standard visual acuity shift were –0.36 ± 0.19 D, 95% confidence interval [–0.40 to –0.33], range –0.88 to 0.13, and 0.20 ± 0.07 LogMAR, 95% confidence interval [0.19 to 0.21], range 0.02 to 0.38, respectively, indicating a decline of two lines in visual acuity under mesopic conditions. Overall, the four theorized dimensions of the Night Driving Questionnaire showed adequate and significant correlations among them, but did not show significant correlations with the criterion variables night refraction and standard visual acuity shift. The whole set of correlations between the NDQ factors and both criterion variables can be found in Table 6.

**Table 6:** Non-parametric correlations (Spearman’s *rho*, 2-tailed) between study factors and criterion variables.

Variable		M	SD		F2	F3	F4	CV^a^	CV^b^
F1	Quality of far vision	3.56	0.58	*rho*	–0.420^**^	–0.309^**^	–0.254^**^	–0.070	0.004
				Sig.	<0.001	<0.001	0.006	0.455	0.966
F2	Difficulties perceiving with central vision	2.44	0.69	*rho*	1.000	0.753^**^	0.733^**^	–0.071	–0.063
				Sig.	--	<0.001	<0.001	0.452	0.500
F3	Difficulties perceiving with peripheral vision	2.01	0.76	*rho*		1.000	0.707^**^	–0.115	–0.006
				Sig.		--	<.001	0.222	0.947
F4	Difficulties perceiving with the interaction of central and peripheral vision	1.83	0.78	*rho*			1.000	–0.158	–0.012
				Sig.			--	0.092	0.898
CV^a^	Night myopia shift	–0.36	0.19	*rho*				1.000	–0.269^**^
				Sig.				--	0.004
CV^b^	Visual acuity shift	0.20	0.07	*rho*					1.000
				Sig.					--

*Notes for the table:* Descriptive values are presented in a scale from 1 (less performance/difficulty) to 5 (more performance/difficulty) or in diopters or logMAR units of change between photopic and mesopic evaluation; ^a,b^ Introduced as a criterion variable (CV). ** The correlation is significant at the *p* < .01 level (2-tailed).

### 3.3. Sex-, age-, and night myopia-based differences

Males self-reported significantly fewer difficulties in perceiving with central and peripheral vision and the combination of both (all *p* ≤ 0.011, η^2^ = 0.055–0.113), as well as suggestive evidence of better quality of far vision, although with a small effect size (*p* = 0.056, η^2^ = 0.032). However, nonsignificant differences between sexes were encountered in sight parameters (refraction shift [night myopia] and standard visual acuity shift [all *p* ≥ 0.167, η^2^ ≤ 0.017]). Descriptive and inferential results can be seen in Table 7.

**Table 7:** Sex-based differences in the NDQ factor (F) scores and criterion variables (CV).

NDQ Factor	Group	N	M^a^	SD^b^	*p*-value (2-tailed)	Effect size (η^2^)
F1. Quality of far vision	Male	54	3.67	0.61	0.056	0.032
	Female	61	3.46	0.54		
F2. Difficulties perceiving with central vision	Male	54	2.24	0.69	**0.003**	0.078
	Female	61	2.63	0.64		
F3. Difficulties perceiving with peripheral vision	Male	54	1.82	0.68	**0.011**	0.055
	Female	61	2.17	0.79		
F4. Difficulties perceiving with the interaction of central and peripheral vision	Male	54	1.56	0.57	**<0.001**	0.113
	Female	61	2.08	0.86		
CV. Night myopia shift	Male	54	−0.35	0.21	0.486	0.004
	Female	61	−0.37	0.18		
CV. Visual acuity shift	Male	54	0.21	0.07	0.167	0.017
	Female	61	0.19	0.06		

*Notes for the table:* Values are presented in a scale from 1 (less performance/difficulty) to 5 (more performance/difficulty), in diopters or logMAR units of change between photopic and mesopic evaluation. Significant values are presented in bold font.

^a^mean; ^b^ standard deviation.

Moreover, a significant main effect of age was encountered on the self-reported quality of far vision (*p* = 0.006, η^2^ = 0.085), but not in the rest of the factors or sight parameters (*p* ≥ 0.260, η^2^ ≤ 0.023). Post-hoc comparisons revealed that young adults reported significantly worse quality of far vision compared to adults (*p* = 0.015) and older adults (*p* = 0.018), while no significant difference was found between adults and older adults (*p* = 0.733). Descriptive results and post-hoc tests can be found in Table 8.

**Table 8:** Age-based differences in the NDQ factor (F) scores and criterion variables (CV).

NDQ Factor	Group	N	M^a^	SD^b^	Groups compared	*p*-value (2-tailed)
F1. Quality of far vision	1 Young adults^c^	35	3.31	0.52	1–2	**0.015**
	2 Adults^d^	61	3.64	0.58	1–3	**0.018**
	3 Older adults^<<^	19	3.75	0.54	2–3	0.733
F2. Difficulties perceiving with central vision	1 Young adults	35	2.58	0.72	1–2	0.562
	2 Adults	61	2.42	0.68	1–3	0.252
	3 Older adults	19	2.26	0.66	2–3	0.633
F3. Difficulties perceiving with peripheral vision	1 Young adults	35	2.10	0.72	1–2	0.698
	2 Adults	61	1.98	0.74	1–3	0.800
	3 Older adults	19	1.95	0.90	2–3	0.992
F4. Difficulties perceiving with the interaction of central and peripheral vision	1 Young adults	35	1.90	0.85	1–2	0.886
	2 Adults	61	1.83	0.71	1–3	0.773
	3 Older adults	19	1.74	0.86	2–3	0.914
CV. Night refraction shift	1 Young adults	35	–0.38	0.19	1–2	0.861
	2 Adults	61	–0.36	0.20	1–3	0.646
	3 Older adults	19	–0.33	0.19	2–3	0.851
CV. Visual acuity shift	1 Young adults	35	0.20	0.19	1–2	0.999
	2 Adults	61	0.20	0.06	1–3	0.327
	3 Older adults	19	0.22	0.07	2–3	0.283

*Notes for the table:* Values are presented in a scale from 1 (less performance/difficulty) to 5 (more performance/difficulty) or in diopters or logMAR units of change between photopic and mesopic evaluation.

Significant values are presented in bold font.

^a^mean; ^b^ standard deviation; ^c^ between 18 and 25 years of age; ^d^ between 26 and 50 years of age; ^e^ between 50 and 65 years of age.

Finally, there were non-significant differences in the questionnaire responses between the participants with less pronounced night myopia ≥ –0.38 D and those with more pronounced night myopia < –0.38 D (all *p* ≥ 0.242, η^2^ ≤ 0.012). As expected, there were differences in the sight parameters refraction shift and visual acuity shift (*p* ≤ 0.018, η^2^ ≥ 0.048). These results are depicted in detail in Table 9, splitting the sample participants according to their night refraction shift values.

**Table 9:** Night refraction shift-based differences in the NDQ factor (F) scores and criterion variables (CV).

NDQ Factor	Night refraction shift	N	M^a^	SD^b^	*p*-value	Effect size (η^2^)
F1. Quality of far vision	≥ –0.38 diopters	68	3.56	0.57	0.976	<0.001
	<–0.38 diopters	47	3.56	0.61		
F2. Difficulties perceiving with central vision	≥ –0.38 diopters	68	2.46	0.62	0.768	0.001
	<–0.38 diopters	47	2.42	0.79		
F3. Difficulties perceiving with peripheral vision	≥ –0.38 diopters	68	2.08	0.74	0.242	0.012
	<–0.38 diopters	47	1.91	0.78		
F4. Difficulties perceiving with the interaction of central and peripheral vision	≥ –0.38 diopters	68	1.88	0.76	0.512	0.004
	<–0.38 diopters	47	1.78	0.81		
CV. Night refraction shift	≥ –0.38 diopters	68	–0.49	0.12	**< 0.001**	0.649
	<–0.38 diopters	47	–0.17	0.11		
CV. Visual acuity shift	≥ –0.38 diopters	68	0.21	0.07	**0.018**	0.048
	<–0.38 diopters	47	0.18	0.06		

*Notes for the table:* Values are presented in a scale from 1 (less performance/difficulty) to 5 (more performance/difficulty) or in diopters or logMAR units of change between photopic and mesopic evaluation. Significant values are presented in bold font; ^a^ mean; ^b^ standard deviation. Night refraction shift: ≥ –0.38 diopters (less pronounced) and <–0.38 diopters (more pronounced).

## 4. Discussion

This study aimed to psychometrically validate the Night Drive Questionnaire (NDQ) and evaluate its potential for early detection and characterization of night myopia. The main finding, which supports the first hypothesis, is that the questionnaire demonstrates adequate psychometric validity and reliability for measuring self-reported quality of far vision and the difficulties experienced during night driving related to central, peripheral, or mixed vision. In contrast with the second hypothesis, while the instrument is valid and structurally adequate, it is not effective for detecting and characterizing night myopia. Therefore, night myopia should be assessed using clinical procedures, such as those validated by Gené-Sampedro et al. [15], until more scientific evidence becomes available.

Hereunder, the discussion is developed attending, first, to the structural features of the questionnaire and the responses given by both sexes and age groups (young adults, adults, and older adults). Second, we will discuss potential reasons for the NDQ not being useful to detect and characterize night myopia, and we will suggest alternative uses for this new questionnaire.

### 4.1. Psychometric properties of the NDQ

The NDQ showed the best psychometric outcomes when a four-factor adjusted model was implemented (F1: Quality of far vision, F2: Difficulties perceiving with central vision, F3: Difficulties perceiving with peripheral vision, F4: Difficulties perceiving with the interaction of central and peripheral vision). Validity and reliability indexes were all appropriate according to statistical standards [39–41], with ordinal indexes (TLI, NFI, IFI, and CFI) above 0.879, alpha coefficients (α) above 0.700, CRI above 0.912, and item factorial weights (λ) above 0.483. Three of the four factors showed appropriate convergent and discriminant validity. Only Factor 2 did not fully satisfy the criteria; however, considering the rest of the reliability items (e.g., CRI ≥ 0.70), convergent and discriminant validity can be accepted [42]. The analysis of inter-item and corrected item–total correlations confirmed the internal homogeneity of all four factors. Although in Factor 2 Item NDQ7 displayed a lower CITC and its removal would have slightly increased α, it was retained due to its conceptual relevance. The theoretical relevance of including this item lies in the potential effect of road lighting in modulating discomfort glare, which may generate a negative psychological response—mental fatigue, headache, and tension—and reduce attention and driving safety [43]. Additionally, street lighting allows drivers to predict the geometry of the road and increases object detection at long distances [44]. Therefore, including this item in the final version of the questionnaire can help potentially detect issues with road lighting in specific areas or individual visual contexts. In summary, these findings reinforce the psychometric robustness of the questionnaire, balancing statistical reliability with content validity.

Apart from these satisfactory psychometric indexes, the four factors formed made theoretical sense in terms of the importance of far vision, central, peripheral, and mixed vision for each of the situations presented in the items, especially at night [13,45,46], which justifies retaining them for this initial validation. The results indicate that the instrument effectively characterizes drivers’ perceived difficulties in common situations encountered during night driving. Specifically, the psychometric structure of the questionnaire allows for a distinction between self-reported quality of far vision, as well as difficulties experienced with central vision, peripheral vision, and the interaction of central and peripheral vision.

The significant and relatively high correlations are hypothetically plausible, given that defects in distance vision can lead to driving difficulties [6]. This includes challenges such as reading highway signage at distances that are considered safe for making vehicle control decisions [13]. Moreover, impairments in both central and peripheral vision (and their combination) also result in driving difficulties [16,47]. In our study, participants who reported better quality in their far vision experienced fewer driving difficulties. Conversely, subjects who had more trouble with their central vision also reported greater difficulties with peripheral and mixed vision. From a statistical perspective, the first factor, “Quality of Far Vision,” should be treated differently from the other three factors of the NDQ in inferential analysis that depend on directional results. This is because Factor 1 reflects positive outcomes (quality of vision), while the other three factors represent negative outcomes (difficulties during night driving). The practical potential of the NDQ and its ability to yield different results based on the respondent’s sex, age, and refraction status are discussed in the following sections.

### 4.2. Known-groups analyses: sex-, age-, and night myopia shift-based differences

Using the NDQ to assess group differences, males self-reported significantly fewer difficulties with night-driving and indicated a trend of better far vision quality; however, the effect sizes for these differences were small to moderate. In contrast, there were no differences between sexes in the measured variables, such as refractive shift and visual acuity shift, where self-report perception does not influence the outcomes. This suggests that sex differences in visual function are heterogeneous across studies, with some research indicating better visual function for females [4] and other studies supporting males [3,48]. Additionally, previous research shows that women are more likely to self-report visual impairment [49] and may restrict their night driving due to perceived difficulties [4]. Therefore, the differences between sexes identified in our study may not accurately reflect actual disparities in driving difficulties. A practical implication of these findings is that sex should be considered when analyzing night driving challenges related to vision.

The analysis revealed no significant differences between age groups in most of the examined variables. However, a significant effect of age was found in the self-reported quality of far vision (*p* = 0.006, η^2^ = 0.085), with post-hoc comparisons showing that young adults reported significantly worse far vision quality compared to adults (*p* = 0.015) and older adults (*p* = 0.018), and no significant difference between adults and older adults (*p* = 0.733). This finding may seem counterintuitive, as it could contrast with previous research indicating that self-reported driving difficulties and tendency to self-restrict nighttime driving increase in individuals over 65 years of age [4,50]. However, it is important to note that our sample included participants with a maximum age of 63 years, and, therefore, these increased difficulties in individuals over 65 years could not be present in our sample. Although older adults are often expected to experience more visual difficulties, their tendency to self-restrict driving in challenging conditions (e.g., at night) and to adopt compensatory strategies may reduce their subjective perception of difficulty. In contrast, younger drivers—who are less likely to self-regulate—may report greater difficulties, despite having better objective visual capacities. This pattern aligns with previous findings suggesting that driving behavior and self-awareness influence perceived visual performance more than age alone [51]. Another potential reason for the reported decline in far vision among young adults may stem from specific characteristics of the sample or other factors, such as young drivers perceiving a higher level of personal risk compared to older drivers [52] or a learning effect observed in more experienced drivers [53].

Older adults have a better tolerance for optical blur than younger adults, making them better at identifying defocused text signs in both daytime and nighttime conditions [54]. At a theoretical level, these changes may be related to optical and neural adaptations that occur with aging [55]. In summary, the results suggest that there are no differences in refraction and visual acuity shifts among the age groups studied (ages 18–63 years) and that the NDQ provides stable outcomes. However, further research is needed to determine if young subjects may have lower quality of far vision when assessed through other visual-related tests beyond refraction and visual acuity, such as contrast sensitivity, discomfort, or disability glare.

Finally, the questionnaire yielded similar results for the groups categorized by the subjects’ level of night myopia (refractive shift). This indicates that individuals with greater night myopia (worse refraction in low-light conditions) do not significantly perceive a decline in the quality of their distance vision or experience increased vision-related difficulties when driving at night. This finding aligns with prior research suggesting that refraction shift is not the primary visual function outcome associated with night driving difficulties; neural complex visual processing linked to light environment may be implied [6].

It is important to note that a previous study indicated that drivers with night myopia of> |0.75| D were more likely to be involved in nighttime driving crashes [7] and we only had one subject with night myopia > |0.75| D and three with night myopia = |0.75| D. The lower representation of participants with values ≥ |0.75| reflects the characteristics of the general population sample available to us. Unlike previous studies that may have applied specific inclusion criteria or recruited targeted populations, our study analyzed a naturally occurring cohort. This can be confirmed by the night myopia range of our study, which was –0.88 and 0.13 D and falls within the range –4.0 to 0.4 D suggested by previous literature [7]. Therefore, the distribution of values observed in our sample accurately represents real-world data, without artificial selection or stratification. Consequently, it is possible that our participants did not report the same level of difficulty as individuals with greater night myopia might have. Future studies should assess whether self-reported night driving difficulties, measured by the NDQ, differ between subjects with night myopia above and below |0.75| D. The implications of these findings are discussed in the following section.

### 4.3. Not effective for measuring night myopia, but… important for driving safety?

While the NDQ was originally developed to capture self-perceived visual difficulties under low-light conditions, our findings indicate that it does not directly correlate with objectively measured night myopia in a population with relatively low levels of night myopia. None of the factors in the questionnaire showed a correlation with night myopia and visual acuity shift. Additionally, there were non-significant differences in the NDQ answers between participants with less pronounced night myopia (refractive shift ≥ –0.38 D) and those with more pronounced night myopia (refractive shift < –0.38 D). These null results suggest that the NDQ may reflect a broader spectrum of subjective visual discomfort, including glare sensitivity, contrast perception, and adaptation to mesopic luminance levels, rather than refractive error alone. According to our findings, a previous study [7] encountered nonsignificant differences in visual complaints between participants with high night myopia (≥ 0.75 D) and those with milder night myopia (< 0.75 D). It is important to note that nearly all subjects from our study had night myopia below the cutoff of |0.75| D; night myopia above this cutoff is considered to pose risks for road safety outcomes [7]. The NDQ might be able to differentiate between drivers with night myopia above or below |0.75| D. However, caution should be applied since we did not measure this, and research on the visual function critical levels for safe driving is limited [56]. For example, the extent of peripheral vision loss that is incompatible with safe driving remains unclear, as compensatory abilities can vary significantly among individuals [16]. Future studies should explore the performance of the NDQ in drivers with higher degrees of night myopia and investigate its predictive value for real-world visual difficulties and safety outcomes (e.g., crashes). Additionally, the use of the NDQ in combination with objective measures may help delineate distinct visual phenotypes affecting nighttime driving performance.

The lack of association between self-reported night-driving difficulties and night myopia may be attributed to other visual function parameters that impact driving performance beyond just shifts in refraction or visual acuity. Factors such as photopic and mesopic contrast sensitivity, along with the disability glare index, are more closely linked to visual challenges experienced during night driving [57]. In fact, contrast sensitivity is a strong predictor of driving outcomes for adults and older adults with normal vision or moderate peripheral field loss [45,58]. Therefore, while the questionnaire used to assess drivers’ self-reported quality of far vision and nighttime visual difficulties is valid and reliable, future research should explore its potential for identifying other critical visual function parameters for safe driving, like contrast sensitivity and disability glare [45,57]. The NDQ clinical relevance may lie in helping to identify individuals who report functional visual difficulties under low-light conditions, even in the absence of clinically significant refractive findings. Although not a diagnostic tool, the NDQ may serve as a complementary screening instrument that prompts more detailed clinical assessments and guides early, targeted interventions.

### 4.4. Limitations and future research

Although all the procedures have been carefully designed and conducted, several limitations should be listed. First, this study did not include direct comparisons with other similar questionnaires (e.g., VND-Q), test–retest reliability, or Rasch analysis, which should be addressed in future research. Regarding the known-groups analysis, the hypothesis that it may detect night myopia (concurrent validity) has been dismissed, which can be considered negative results [59]; however, the psychometric construct validity of the questionnaire has been tested and systematically endorsed through different tests, coefficients, and empirical insights. Considering that our study included mild cases of night myopia, these findings hinder the ability of the NDQ to detect subtle changes during early stages of visual deterioration.

In this context, this study can serve as a foundation for future applications of the questionnaire among drivers with higher night myopia or those with specific conditions (e.g., early glaucoma or age-related macular degeneration). Although participants reported general information about their estimated driving frequency, this information was not considered in the analysis. Future research should examine how different driving environments (e.g., urban vs. rural roads, road lit conditions) and driving distances interact with night vision and contribute to perceived visual difficulties while driving at night.

Moreover, it is important to note that our subjects exhibited night myopia values ranging from –0.88 to 0.13 D, with most of them (95% confidence interval) falling between –0.40 and –0.33 D, which limits the questionnaire’s ability to characterize more severe cases of night myopia. The presence of a mild hyperopic shift may reflect individual variability in accommodative behavior, small measurement fluctuations, or retinal/neural adaptation mechanisms under scotopic conditions. Although this phenomenon has been occasionally reported in the literature, its prevalence remains low, as also observed in our sample. This fact is likely due to our sample being randomly selected. Due to the small number of cases, a separate statistical analysis of this subgroup was not feasible or meaningful. Therefore, our analysis remained focused on the predominant trend of night myopia, which is consistent with the main objective of the study: to validate the NDQ concerning night vision difficulties. This is an interesting avenue for research regarding further studies using tools such as the NDQ in convenience samples, including driving populations with other specific features.

Finally, while our study’s sample is balanced in terms of gender, it is not balanced across age groups. However, this does not necessarily imply a strong bias, as previous research indicates that night driving difficulties tend to remain relatively stable in healthy adults below 65 years [4,50]. Like similar questionnaires previously developed, the subjective nature of the NDQ means that the results depend on the individual’s self-perception, which may not accurately reflect actual functional impairment [60–62]. Therefore, future versions of the NDQ should include a qualitative question about adaptive behaviors to counteract the reported nighttime driving difficulties. Lastly, this tool does not replace objective evaluation and should therefore be considered as a complementary measure to clinical vision tests relevant for driving.

## 5. Conclusion

The main conclusion is that the Night Drive Questionnaire is valid and reliable for assessing self-perceived quality of far vision and the difficulties encountered during night driving related to central and peripheral vision, as well as their combination. However, this questionnaire does not effectively characterize clinically measured night myopia or shifts in visual acuity. Therefore, it would be convenient to clinically evaluate drivers’ refraction and visual acuity shift, rather than using only a questionnaire, for global sight assessments. Future studies should investigate whether the NDQ can better characterize other visual function parameters that are more directly associated with night driving challenges.

## Supporting information

S1 File Raw Data [S1_Database.zip]. (SAV)

S2 File Root Questionnaire [S2_Root_Questionnaire.pdf]. (PDF)
