# Supplementary material for: Self-perceived far vision quality and night driving difficulties are not related to night myopia or visual acuity
Source: PLoS One. 2026 Feb 5;21(2):e0339372. doi: 10.1371/journal.pone.0339372 (PMC12875446; doi:10.1371/journal.pone.0339372)
Supplement: S2 File — (PDF) [file pone.0339372.s002.pdf]

S2 File. Root Questionnaire - Researcher Form

Question		Possible answers
**General driving questions**		
What type of vehicle do you usually drive? (multiple choice question)		Car<br>Motorbike<br>Van<br>Truck<br>Bus
How many years ago did you start driving?		Less than 1 years<br>Between 1 and 5 years<br>Between 5 and 10 years<br>Between 10 and 15 years<br>More than 15 years
How many days per week do you usually drive during the night (between 7:00 pm and 7:00 am)?		Less than 2 days<br>2 or 3 days<br>4 or 5 days<br>6 or 7 days
How many minutes per day do you usually drive during the night (between 7:00 pm and 7:00 am)?		Less than 20 min<br>Between 20 min and 1 hour<br>Between 1 and 2 hours<br>Between 2 and 3 hours<br>More than 3 hours
How many kilometres do you usually drive during the night (between 7:00 pm and 7:00 am)?		Less than 20 km<br>Between 20 and 50 km<br>Between 50 and 80 km<br>Between 80 and 100 km<br>More than 100 km
In what areas do you usually drive during the night (between 7:00 pm and 7:00 am)?		Urban areas<br>Highways (generally well illuminated)<br>Secondary roads (generally not well illuminated)
How frequently?<br>- Urban areas<br>- Highways<br>- Secondary roads		Never<br>Rarely<br>Sometimes<br>Frequently<br>Most of time
**Night Driving Questionnaire**		
NDQ1	How would you rate your distance vision performance (with glasses or contact lenses, if you typically use them)?	Very poor<br>Poor<br>Acceptable<br>Good<br>Excellent

NDQ2	How would you rate your distance vision performance during day driving?	Very poor<br>Poor<br>Acceptable<br>Good<br>Excellent
NDQ3	How would you rate your distance vision performance during night driving (between 19:00 and 7:00 hs)?	Very poor<br>Poor<br>Acceptable<br>Good<br>Excellent
NDQ4	Do you perceive difficulty in reading traffic signs or panels on the road during night driving (between 19:00 and 7:00 hs)?	No difficulty<br>Light difficulty<br>Moderate difficulty<br>Increased difficulty<br>Extreme difficulty
NDQ5	Do you perceive difficulty in seeing the road when it rains during night driving (between 19:00 and 7:00 hs)?	No difficulty<br>Light difficulty<br>Moderate difficulty<br>Increased difficulty<br>Extreme difficulty
NDQ6	Do you perceive difficulty in seeing the road due to the lights of cars that come from the front during night driving (between 19:00 and 7:00 hs)?	No difficulty<br>Light difficulty<br>Moderate difficulty<br>Increased difficulty<br>Extreme difficulty
NDQ7	Do you perceive difficulty in seeing the road due to the street/road lighting during night driving (between 19:00 and 7:00 hs)?	No difficulty<br>Light difficulty<br>Moderate difficulty<br>Increased difficulty<br>Extreme difficulty
NDQ8	Do you perceive difficulty in detect pedestrians or animals (or other moving objects) on the road during night driving (between 19:00 and 7:00 hs)?	No difficulty<br>Light difficulty<br>Moderate difficulty<br>Increased difficulty<br>Extreme difficulty
NDQ9	Do you perceive difficulty in seeing obstacles (bumps, potholes, roadsides) on the road during night driving (between 19:00 and 7:00 hs)?	No difficulty<br>Light difficulty<br>Moderate difficulty<br>Increased difficulty<br>Extreme difficulty
NDQ10	Do you perceive difficulty in seeing (with your peripheral vision) obstacles that appear suddenly in your field of vision while you are looking forward during	No difficulty<br>Light difficulty<br>Moderate difficulty<br>Increased difficulty<br>Extreme difficulty

	night driving (between 19:00 and 7:00 hs)?	
NDQ11	Do you perceive difficulty in seeing traffic lights or beacons on the road during night driving (between 19:00 and 7:00 hs)?	No difficulty<br>Light difficulty<br>Moderate difficulty<br>Increased difficulty<br>Extreme difficulty
NDQ12	Do you perceive difficulty in estimating the distance to a road exit or detour during night driving (between 19:00 and 7:00 hs)?	No difficulty<br>Light difficulty<br>Moderate difficulty<br>Increased difficulty<br>Extreme difficulty
NDQ13	Do you perceive difficulty in estimating the distance to a nearby vehicle during night driving (between 19:00 and 7:00 hs)?	No difficulty<br>Light difficulty<br>Moderate difficulty<br>Increased difficulty<br>Extreme difficulty
	**Additional questions**	
21	Do you consider you adjust/modify your driving during the night compared to during the day?	Yes<br>No
22	What adaptations or modifications do you usually adopt?	Slightly close the eyes or frown to see better<br>Place the hand in front of the eyes<br>Reduce the speed<br>Reduce driving time<br>Limit the driving to well-illuminated areas<br>Limit the driving to well-known areas<br>Turning the head frequently to watch the sides<br>Other

Hs: hours; min: minutes; km: kilometers.
